# Enhancing the compost maturation of swine manure and rice straw by applying bioaugmentation

**DOI:** 10.1038/s41598-021-85615-6

**Published:** 2021-03-17

**Authors:** Wei-Kuang Wang, Chih-Ming Liang

**Affiliations:** Department of Environmental Engineering and Science, Feng Chia University, Taichung, 40724 Taiwan

**Keywords:** Microbiology techniques, Environmental biotechnology, Industrial microbiology

## Abstract

Microorganisms capable of decomposing cellulose, xylan, starch and protein were individually isolated from swine manure compost and soil in this study. The correlations with pH, carbon source concentration, C/N ratio and enzyme activity among these isolated microorganisms were also investigated. Furthermore, the effect of additional inoculation in the compost was studied by measuring variations in the C/N ratio, enzyme activity and compost maturation rate. The inoculated microorganisms used in this study included four bacterial isolates and one commercial microorganism *Phanerochaete chrysosporium*. The results indicated that the isolated *Kitasatospora phosalacinea* strain C1, which is a cellulose-degraded microorganism, presented the highest enzyme activity at 31 ℃ and pH 5.5, while the C/N ratio was 0.8%. The isolated xylan-degraded microorganism *Paenibacillus glycanilyticus* X1 had the highest enzyme activity at 45 ℃ and pH 7.5, while the C/N ratio was 0.5%. The starch-degraded microorganism was identified as *Bacillus licheniformis* S3, and its highest enzyme activities were estimated to be 31 ℃ and pH 7.5 while the C/N ratio was 0.8%. The highest enzyme activity of the protein-degraded microorganism *Brevinacillus agri* E4 was obtained at 45 ℃ and pH 8.5, while the C/N ratio was 1.0%. The rate of temperature increase in the compost inoculated with *P. chrysosporium* was only higher than that of the compost without inoculation, and its compost maturation level was also lower than that of other composts with additional inoculation. The optimal initial C/N ratio of the compost was 27.5 and the final C/N ratio was 18.9. The composting results also indicated that the secondary inoculation would benefit compost maturation, and the lowest final C/N ratio of 17.0 was obtained.

## Introduction

Compost processes have been extensively applied to treat agricultural and livestock waste. According to white papers published by the Council of Agriculture of Executive Yuan in Taiwan, swine manure and rice straw are the main agricultural wastes. The rice harvest area in Taiwan is approximately 270 thousand hectares, which produces 1.62 million tons of rice straw. There are 6 million swine in Taiwan, which produce 11 thousand tons of manure each year. Rice straw is a lignocellulose that is slowly biodegradable^1–4^. Rice straw contains 90% organic matter, which includes 24.7% cellulose, 20.6% semicellulose and 7.7% lignin. The carbon and nitrogen contents of the rice straw were 53.0% and 0.7%, respectively. Swine manure contains 75.0% organic matter and 10.2% cellulose and semicellulose. Previous research has focused on the effect of inoculation during composting^5–10^. Among all the microbial strains capable of decomposing lignocellulose, the fungus *Phanerochaete chrysosporium* has been proven to have an excellent ability to degrade lignin and a wide variety of aromatic compounds^8,11^. However, previous studies primarily performed inoculation with *P. chrysosporium* during the initial phase of composting; thus, the effect of inoculation at different composting stages has not been clarified.

After the waste rice straw and swine manure are reused and treated via the compost process, the mature compost can be used as an agricultural fertilizer. Composting refers to the use of microorganisms, such as bacteria (e.g., *Acinetobacteria*) and fungi, to decompose organic matter and transfer it into humic matter under certain environmental conditions. Heat is generated by the biological transformation, and the rising temperature promotes biodegradation and stabilization of agricultural waste. Environmental factors, such as temperature, pH, moisture content and nutrients, affect the composting process^12–17^. The process of decomposing organic matter through the action of microorganisms is mainly affected by the oxygen and moisture contents, although temperature, nutrients and pH are also important factors influencing the composting process. Changes in temperature are related to the activity of microorganisms and the carbon and nitrogen contents influence the growth and activity of microorganisms. Organic matter represents a source of carbon and energy required for the growth of microorganisms and nitrogen required for cell synthesis. Although phosphorus and sulfur are also important for the growth of microorganisms, few studies have mentioned the effects of phosphorus and sulfur on composting^18^.

The stages of the composting process and the usefulness of compost as an organic amendment are determined by the microbial activity^19–22^. Related enzymes secreted by composting microorganisms play an important role in transforming organic matter into humic matter. These enzymes, such as cellulase, xylanase, amylase and proteinase, are responsible for breaking down complicated organic compounds and producing simple water-soluble compounds^20,23,24^. Analyses of the presence and activities of related enzymes during composting might provide evidence for further understanding and developing the waste biodegradation processes^11,23^. Humic matter is a high molecular weight compounds that can improve the ion exchanged capability and the nutrient composition of soil and help modify the soil structure. Increasing temperature restructures the microbial community, breaks down toxic compounds, and reduces pathogens, worm ovum and grass seeds. Normally, compost requires 2–4 months to mature. If optimal microbes from swine manure and rice straw could be inoculated into composts, then the maturation time could be shorten and waste could be transformed into useful material to reach the goal of circular economy.

In this study, several bacterial isolates capable of decomposing cellulose, xylan, starch and protein were applied for bioaugmentation. The cellulase, xylanase, amlyase and proteinase activities were measured to evaluate the feasibility of bioaugmentation for improving the composting process. The aim of this study was to understand the effect of various conditions on the composting process and explore the feasibility of bioaugmentation for improving compost mature maturation.

## Materials and methods

### Experimental set-up

Three stages of experiments were designed in this research. In the first stage, microorganisms with cellulose-, xylene-, starch- and protein-degrading abilities were isolated. In the second stage, the effects of pH, temperature, and carbon source concentration on the microbial growth and extracellular enzyme activities of each bacterial isolate were explored. In the third stage, the compost was inoculated with the bacterial isolates and fungus *P. chrysosporium* to determine the effect of inoculation on the compost maturation rate.

### Microbial strains and medium

The cellulose-degrading and xylan-degrading bacterial strains used in this research were isolated from woodland soil in central Taiwan. Starch-degrading and protein-degrading bacterial strains were isolated from compost in a pig farm located in Yunlin County. The fungal strain *P. chrysosporium* BCRC 36200 (ATCC 24725) was purchased from the Bioresource Collection and Research Centre in Taiwan. Several synthetized media were used in this research. The medium used for screening the cellulose-degrading bacteria contained 5.0 g/L carboxymethyl cellulose (CMC), 1.0 g/L (NH_4_)_2_SO_4_, 1.0 g/L KH_2_PO_4_, 0.2 g/L MgSO_4_·7H_2_O, 5.0 mg/L FeCl_3_·6H_2_O, 0.1 g/L CaCl_2_·2H_2_O and 1.5% (v/v) agar. The medium for isolating the xylan-degrading bacteria was composed of 5.0 g/L xylan, 66.8 mg/L NH_4_Cl, 1.5 mg/L CaCl_2_, 0.2 g/L MgSO_4_·7H_2_O, 0.5 g/L K_2_HPO_4_, 0.5 g/L KH_2_PO_4_, 10.0 mL/L trace elements (trace elements: 0.3 g/L FeSO_4_·7H_2_O, 40.0 mg/L ZnSO_4_·7H_2_O, 58.0 mg/L CoCl_2_, 50.0 mg/L MnSO_4_·H_2_O, 34.0 mg/L Na_2_MoO_4_·2H_2_O, and 50.0 mg/L CuSO_4_·5H_2_O) and 1.5% (v/v) agar. The medium for screening the starch-degrading bacteria contained 5.0 g/L soluble starch, 8.0 g/L nutrient broth, and 1.5% (v/v) agar. The medium for screening protein-degrading bacteria was composed of 10.0 g/L skim milk, 8.0 g/L nutrient broth and 1.5% (v/v) agar. The medium for isolating xylan-degrading bacteria contained 5.0 g/L lignin, 10.0 g/L malt extract, 2.0 g/L yeast extract, 2.0 g/L KH_2_PO_4_, 1.0 g/L MgSO_4_·7H_2_O and 1.5% agar. The bacterial isolates in this study were identified according to their 16S rDNA sequences. The DNA sequencing and identification of the isolated microorganisms in this study were commissioned and carried out by Mission Biotech Co., Ltd., Taiwan.

### Extracellular enzyme activity assessment

For the cellulsase and xylanse assay, the bacterial isolates were streaked onto solid specific media and incubatedat 25 °C for 3 days. To identify the enzyme activity, a two-step visualization process was performed. Congo red (0.1% (v/v)) staining was performed for 5 min, and then the destaining process was carried out by soaking the stained sample in 1.0 M NaCl for 10 min. The enzyme activity and cellulose-degrading ability could be identified by the size of the clear zone on the medium produced by the bacterial isolates^25^. To identify the starch hydrolyase activity, the specific strain was incubated on the synthesized medium at 37 °C for 3 days. Liquid iodine was used for staining for 10 min. The starch-degrading ability could be understood by visualizing the size of the clear zone^26^. To identify the proteinase, the specific strain was incubated onto skim milk medium under 37 °C for 3 days. The protein-degrading ability could also be identified by the size of clear zone on the medium^27^.

### Manure and rice straw-degrading potential of isolated strains

To understand the effects of temperature and pH on the manure and rice straw degradation potential of the isolates, the bacterial isolates were transferred into 100 mL media with different pH values (5.5, 6.5 and 8.5) and, the initial optical density (O.D) value was adjusted to 0.1 ± 0.02 at the wavelength of 600 nm. All the microorganisms used in this study were incubated at various temperatures of 25, 31, 37 and 45 ℃ at 120 rpm (Table 1). The incubation was terminated at 120 h while the microorganisms grew to stationary phase. The temperature and pH that corresponded to the highest activity of the target extracellular enzyme were then selected for further examination. The effect of carbon source concentration on the target enzyme activities was analyzed at carbon source concentrations of 0.2, 0.5 and 0.8%.

**Table 1:** Different temperature and pH conditions for isolates incubation.

Extracellular enzyme	Incubation temperature ( ℃)	pH value
Cellulosase	25	5.5, 6.5, 7.5 and 8.5
Xylanse	31	5.5, 6.5, 7.5 and 8.5
Starch hydrolyase	37	5.5, 6.5, 7.5 and 8.5
Protein hydrolyase	45	and 8.5

### Composting examination

A composting reactor with a volume of 24 L was applied in this study. This reactor was made of high-density polyethylene and equipped with a perforated plate barrier at the bottom, which allowed for the transfer of air influent and water effluent. There was also a channel for air exhaust at the top of this reactor. The reactor was covered by glass fibres to maintain the temperature. The organic matter for composting in this study was a mixture of swine manure and rice straw adjusted to different initial C/N ratios. The swine manure was collected from a swine farm and rice straw was collected from a rice paddy. The organic matter mixtures were then inoculated with four isolated microorganisms and a pure culture of the fungus *P. chrysosporium* purchased from the Bioresource Collection and Research Centre (BCRC) at different composting stages (Table 2). The carbon, nitrogen and moisture contents of the swine manure were 43%, 3% and t 72% respectively, while these contents in the rice straw were 53%, 0.7% and 14%, respectively. All composting experiments were carried out simultaneously and at ambient temperature.

**Table 2:** Inoculation at different composting stages and initial C/N ratio.

Inoculated strains	Composting stage	Initial C/N ratio	Composting testing group
Without inoculation	–	27.5	Group 1
*Phanerochaete chrysosporium*	Mesophilic stage	27.5	Group 2
Isolated cellulose-degrading bacterial strain	Mesophilic stage	27.5	Group 3
Isolated starch-degrading bacterial strain		25	Group 4
Isolated xylan-degrading bacterial strain		30	Group 5
Isolated protein-degrading bacterial strain	Mesophilic stage and cooling stage	27.5	Group 6

### Analytical methods

The temperature of the composting mixture in the composting reactor was monitored every day, and samples were collected during the composting process for further analysis. The total nitrogen (TN) was measured by the Kjeldahl method^28^. The moisture content of the samples was determined after drying at 105 ± 5 ℃ for 2 h. The dried samples were then ground and weighed after cooling to room temperature. Subsequently, the samples were weighed after combustion at 550 ± 50 ℃ for 6 h. The ash content (%Ash) was calculated based on the difference between the weight of samples before and after combustion. The carbon content (%C) could be calculated according to the following formula^29^

$$ \% {\text{C}} = {{\left( {{1}00 - \% {\text{Ash}}} \right)} \mathord{\left/ {\vphantom {{\left( {{1}00 - \% {\text{Ash}}} \right)} {{1}.{8}}}} \right. \kern-\nulldelimiterspace} {{1}.{8}}} $$

In addition, a seedling growing test was carried out using mature compost. A 5 g compost sample was mixed with 100 mL H_2_O and incubated at 60 ℃ for 3 h. The mixture was then filtered with fine cloth paper. Two pieces of clean filter papers were put onto the Petri dish, and 10 mL of filtered liquid was added. Finally, 25 cabbage seeds of *Brassica oleracea* Linn. var. capitata DC. were placed on the surface of the filter paperand incubated at 25 ℃ for cultivation. For the blank test, ddH_2_O was used to replace the filtered liquid. The seedling growing conditions were observed and evaluated after 3 days.

### Measurement of bacterial growth and extracellular enzyme activities

The OD_600_ value was applied to determine bacterial growth in this study. For measuring endocellulase, known as CMCase, a crude enzyme solution was prepared as follows. After mixing 10 g compost with 40 mL 100 mM Tris–HCl buffer (pH 7.5), the solution was incubated at 30 °C for 30 min. Then the solution was centrifuged at 5000 rpm for 15 min. The supernatant was used as the crude enzyme solution^30^. The crude euzyme solution (0.5 mL) was mixed with 0.5 mL of matrix solution (0.2% CMC dissolved in 0.1 M phosphate buffer solution (pH 7)). After incubation at 50 °C and 150 rpm for 30 min, 1 mL of 3,5-dinitrosalicyclic acid (DNS) reagent was added as a colour reagent and incubated at 100 ℃ for 5 min. After cooling to room temperature, the sample was measured at a wavelength of 540 nm with a spectrophotometer (High, 2012). One U (μmol/min) of CMCase was defined as the amount of the enzyme that catalysed the conversion of one micromole of substrate per minute under the specific conditions according to the assay method. In this study, 1 U of CMCase representing 1 μmole glucose/mL was released per min. For xylase activity measurement, 0.5 mL crude enzyme solution and 0.5 mL of matrix solution (0.2% beechwood xylan dissolved in 50 mM sodium citrate buffer solution (pH 6)) were mixed and incubated at 37 °C for 37 min. Then, 1 mL DNS reagent was added as a colour reagent and the mixture was placed at 100 ℃ for 5 min. The mixture was then measured at 540 nm with a spectrophotometer after cooling to room temperature^31^. 1 U of the xylase activity represented 1 μmole xylose/mL was released per min. For starch hydrolyase activity measurement, known as amylase activity measurement, 0.5 mL crude enzyme solution was mixed with 0.5 mL matrix solution (0.2% soluble starch dissolved in 0.1 M phosphate buffer solution (pH 7)) and reacted at 40 °C for 10 min. Then, 1 mL DNS reagent was added and the mixture was placed at 100 °C for 5 min. The absorbance of the sample was measured at a wavelength of 540 nm using a spectrophotometer^32^ after cooling. 1 U of amylase activity represented 1 μmole glucose/mL released per min. Proteinase activity was represented by the protein hydrolase activity. To measure the activity, 0.1 mL crude enzyme solution was mixed with 0.5 mL matrix solution (0.1% casein in phosphate buffer solution), and reacted at 37 °C for 30 min in a water bath. Subsequently, 0.9 mL of 0.3 M trichloroacetic acid was added to the mixture, which was centrifuged at 3000 rpm for 10 min; then, 0.2 mL of the supernatant was mixed with 1.1 mL of lyeand 0.2 mL of 50% Folin-Ciocalteu phenol was added. The mixture was centrifuged at 8000 rpm for 10 min, and the absorbance was measured at 578 nm with a spectrophotometer^32^. In which, 1 U of the proteinase activity represented 1 μmole tyrosine/mL released per min.

## Results and discussion

### Identification and screen of isolated microorganisms

Seven pure cultures capable of degrading cellulose were isolated and referred to as strains C1–C7. The clear zone ratios of pure strains C1–C7 were 3.66, 1.33, 1.5, 2, 1.4, 1.33 and 1.25, which indicated that strain C1 had the highest clear zone ratio and could be the best cellulolytic bacterium. Strain C1 was then identified as *Kitasatospora phosalacinea* C1 after 16S rDNA sequencing. For screening xylan-degrading bacteria, one pure culture was obtained, and its clear zone ratio was 2.35. This isolate was identified as *Paenibacillus glycanilyticus* X1. For starch-degrading bacterial isolation, 11 pure cultures were obtained and assigned as S1–S11. Strain S3 with the highest clear zone ratio of 4.08 was identified as *Bacillus licheniformis* S3. Other isolates of strains E1–E7 were proven to have the protein-degrading abilities, and strain E4 was suggested to have the highest protein-degrading ability and was identified as *Brevibacillus agri* E4. The clear zone ratios of each isolate are presented in Table 3.

**Table 3:** The clear zone ratios of isolates in this study.

Isolates	Colony size (mm)	Clear zone (mm)	Clear zone ratio	Isolates	Colony size (mm)	Clear zone (mm)	Clear zone ratio
C1	6	22	3.66	S6	1.6	3.8	2.36
C2	3	4	1.33	S7	1.65	3.45	2.09
C3	4	6	1.50	S8	0.8	2.7	3.38
C4	2	4	2.00	S9	2.1	3.6	1.71
C5	5	7	1.4	S10	1.5	3.7	2.47
C6	6	8	1.33	S11	1.85	3.55	1.92
C7	8	10	1.25	E1	1.1	2.9	2.63
X1	40	17	2.35	E2	0.6	2	3.33
S1	0.7	2.5	3.57	E3	2.3	2.7	12.17
S2	0.65	2.55	3.92	E4	0.4	1.6	4
S3	0.65	2.65	4.08	E5	0.4	1	2.5
S4	0.55	1.65	3.0	E6	2.4	3	1.25
S5	2.0	3.8	1.9	E7	1	1.8	1.8

### Effects of temperature and pH on the enzyme activities of the isolated bacteria

**Figure 1:**
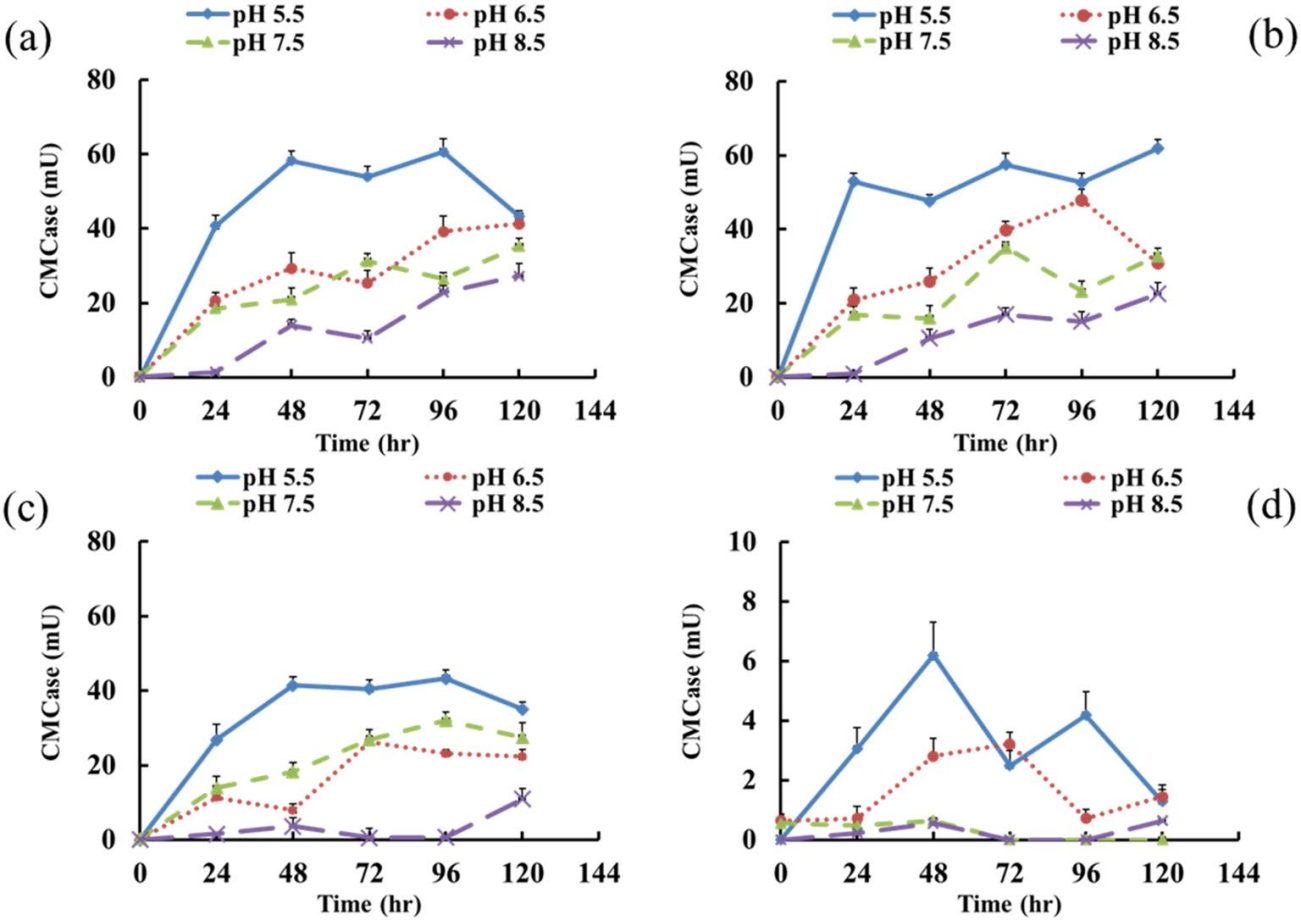
The change of CMCase activity of strain *Kitasatospora phosalacinea* C1 under different temperature and initial pH conditions. (**a**) 25 ℃, (**b**) 31 ℃, (**c**) 37 ℃, (**d**) 45 ℃.

*Kitasatospora phosalacinea* C1 incubated at 31 °C and an initial pH of 5.5 exhibited the best CMCase activity. The average enzyme activity under this condition was 42.84 mU (Fig. 1b), which was approximately 190 times higher than that of 0.24 mU at 5 °C and an initial of pH 8.5 (Fig. 1d). In addition, when the incubation temperature was between 25 and 45 ℃, the best enzyme activity was achieved at a pH of 5.5 (Fig. 1a,c,d). Increasing the incubation temperature led to a decline in CMCase activity. Emtiazi and Nahvi indicated that when *Cellulomonas* sp. was cultured within 1% whey straw at 28 °C and an initial pH of 7.0, and the highest CMCase activity (3 U) was obtained at 72 h. However, the enzyme activity significantly declined after 72 h^33^. Although the results of the CMCase activity under the best environmental conditions in this study were not as good as the highest enzyme activity in the literature, the enzyme activity still slightly increased after 96 h.

**Figure 2:**
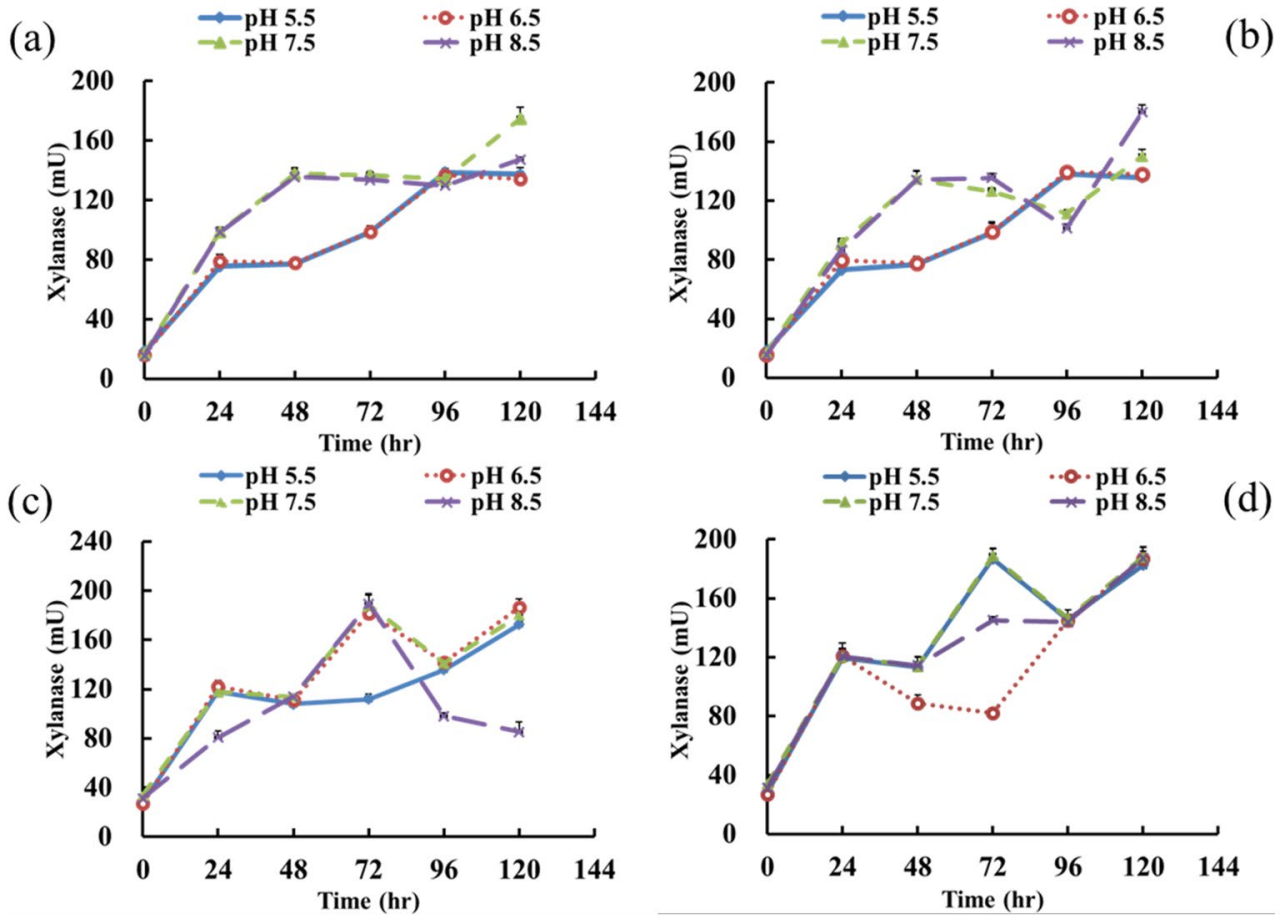
Xylanase activity changes of strain *Paenibacillus glycanilyticus* X1 under different temperature and pH conditions. (**a**) 25 ℃, (**b**) 31 ℃, (**c**) 37 ℃, (**d**) 45 ℃.

The highest average xylanase activity of 131.75 mU was obtained when *P. glycanilyticus* strain X1 was cultured at 45 °C and an initial pH of 7.5 (Fig. 2d). The lowest average xylanase activity of 90.12 mU was obtained when the strain was incubated at 31 °C and an initial pH of 5.5 (Fig. 2b). The highest xylanase activity reached 174.67 mU and 179.91 mU within 120 h when the strain was cultured at 25 ℃ and an initial pH of 7.5 and 31 ℃ and an initial pH of 8.5 (Fig. 2a,b). When the culture temperature increased to 37 ℃ and the initial pH was 8.5, the xylanase activity reached the highest amount of 189.6 mU at 72 h. Furthermore, when the incubation temperature and initial pH were 45 ℃ and 7.5, the highest xylanase activity of 188.53 mU was also observed at 72 h. Both results demonstrated that increasing the incubation temperature resulted in a shortened the time for xylanase to exhibit high activity (Fig. 2c,d). According to the results of this experiment, *P. glycanilyticus* strain X1 could have higher enzyme activity under high temperature. Based on various culture temperatures, *P. glycanilyticus* strain X1 showed no significant difference in xylanase activity. Thus, when *P. glycanilyticus* strain X1 was inoculated into the compost, it could increase the reaction time for degrading xylan. Ko et al. cultivated *Paenibacillus campinasensis* strain BL11 underan initial pH of 8 and various temperatures (25 ℃, 37 ℃ and 50 ℃). The results illustrated that the highest enzyme activity was obtained when the initial pH was 8 and the incubation temperature were 37 ℃ and 50 ℃^34^. Their results were consistent with our results, with higher xylanase activity obtained under neutral and slight alkali conditions and at high to moderate temperatures.

**Figure 3:**
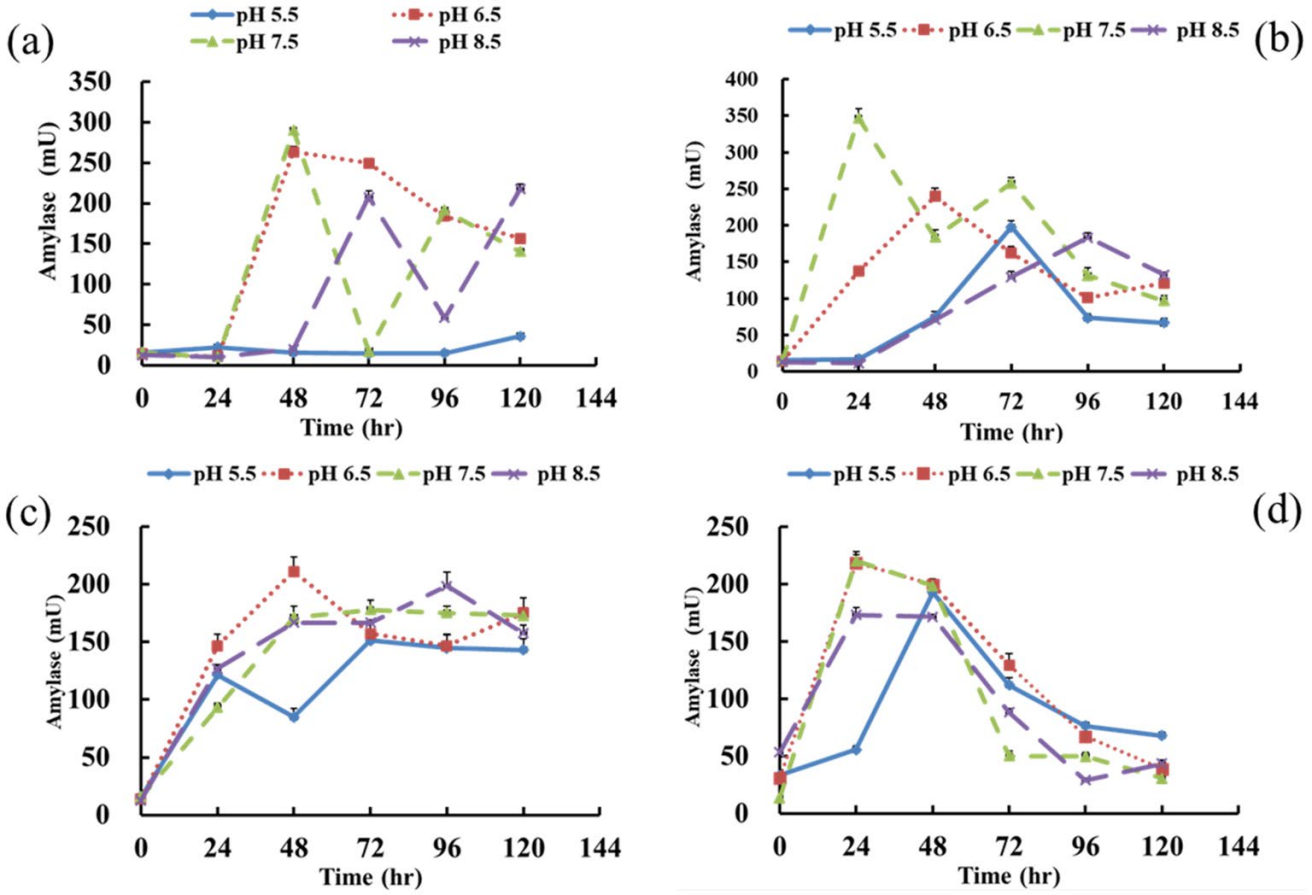
The change of amylase activity of *Bacillus licheniformis* S3 strain under different temperature and pH conditions. (**a**) 25 ℃, (**b**) 31 ℃, (**c**) 37 ℃, (**d**) 45 ℃.

The amylase activity of the starch-degrading *B. licheniformis* strain S3 under different temperatures and different initial pH values is shown in Fig. 3. The results showed that the average amylase activity was lowest when the strain was cultured at an initial pH of 5.5 and four culture temperatures of 25 °C, 31 °C, 37 °C and 45 °C (Fig. 3a–d). The proper conditions for *B. licheniformi*s strain S3 to produce amylase were suggested to be at 31 °C and an initial pH of 7.5. Under this condition, the amylase activity reached 346.74 mU within 24 h and the average enzyme activity was 171.97 mU. The average amylase activity was higher than that under other conditions (Fig. 3b). Ashraf et al. demonstrated that the amylolytic bacteria *Bacillus licheniformis* strain GCBU-8 showed the highest enzyme activity at 48 h when the strain was cultured at 40 °C^35^. In contrast, the results in this study demonstrated that the highest enzyme activity could be obtained at the 24th hour.

**Figure 4:**
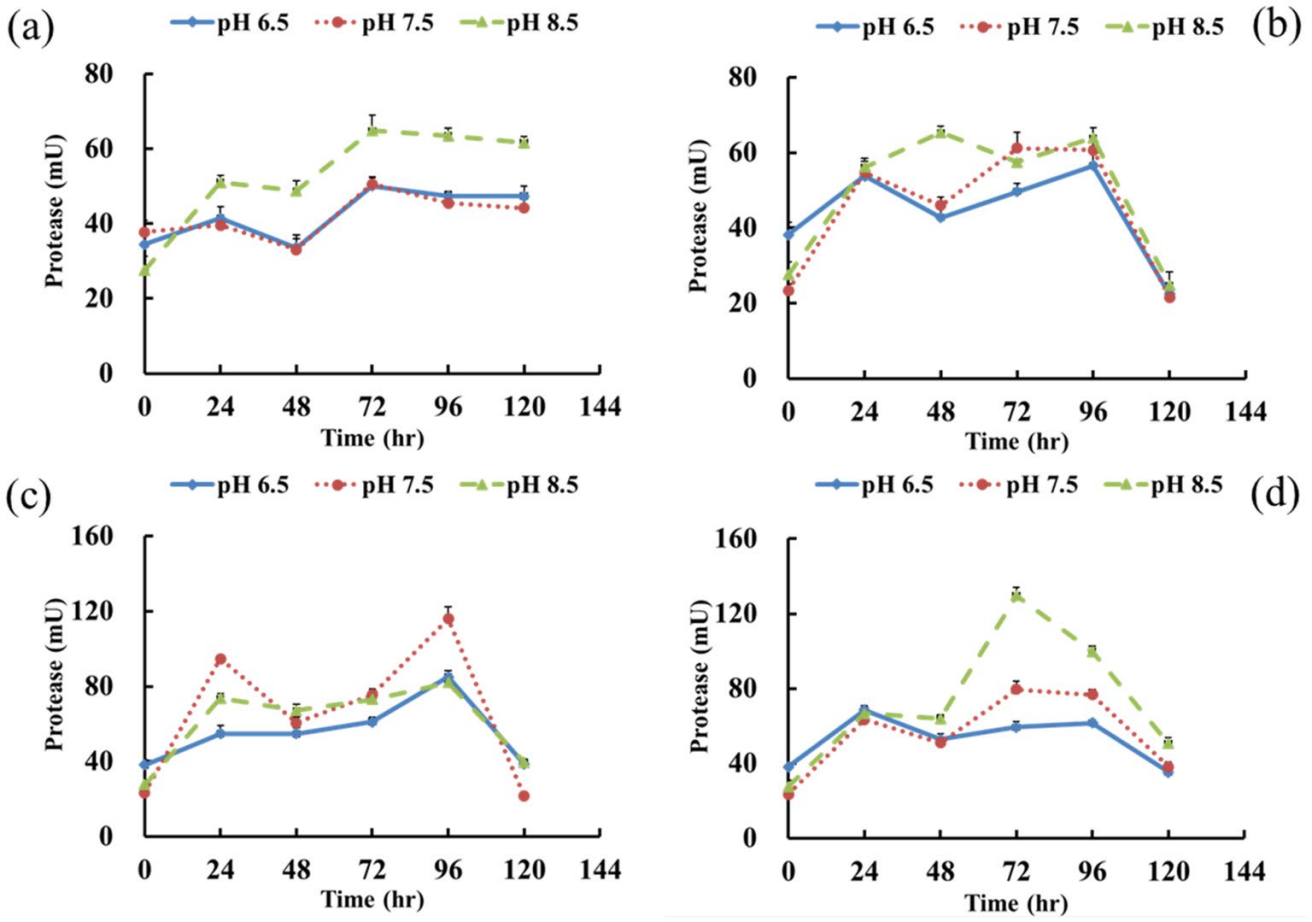
Proteinase activity changes of strain *Brevibacillus agri* E4 under different temperature and pH conditions. (**a**) 25 ℃, (**b**) 31 ℃, (**c**) 37 ℃, (**d**) 45 ℃.

Figure 4 displays the proteinase activity of *B. agri* strain E4 at different temperatures and different initial pH values. With increasing cultivation temperature, the overall proteinase activity of the *B. agri* strain E4 tended to increase. When the initial pH was 8.5, all incubation temperature groups had the best proteinase activities, except for 37 °C. The highest proteinase activity of 129.7 mU was obtained at 45 ℃ and an initial pH was 8.5. The average proteinase activity was 73.05 mU, which was the best cultivation condition for proteinase secretion in this study (Fig. 4d).

### The effect of carbon source concentration on enzyme activities

**Figure 5:**
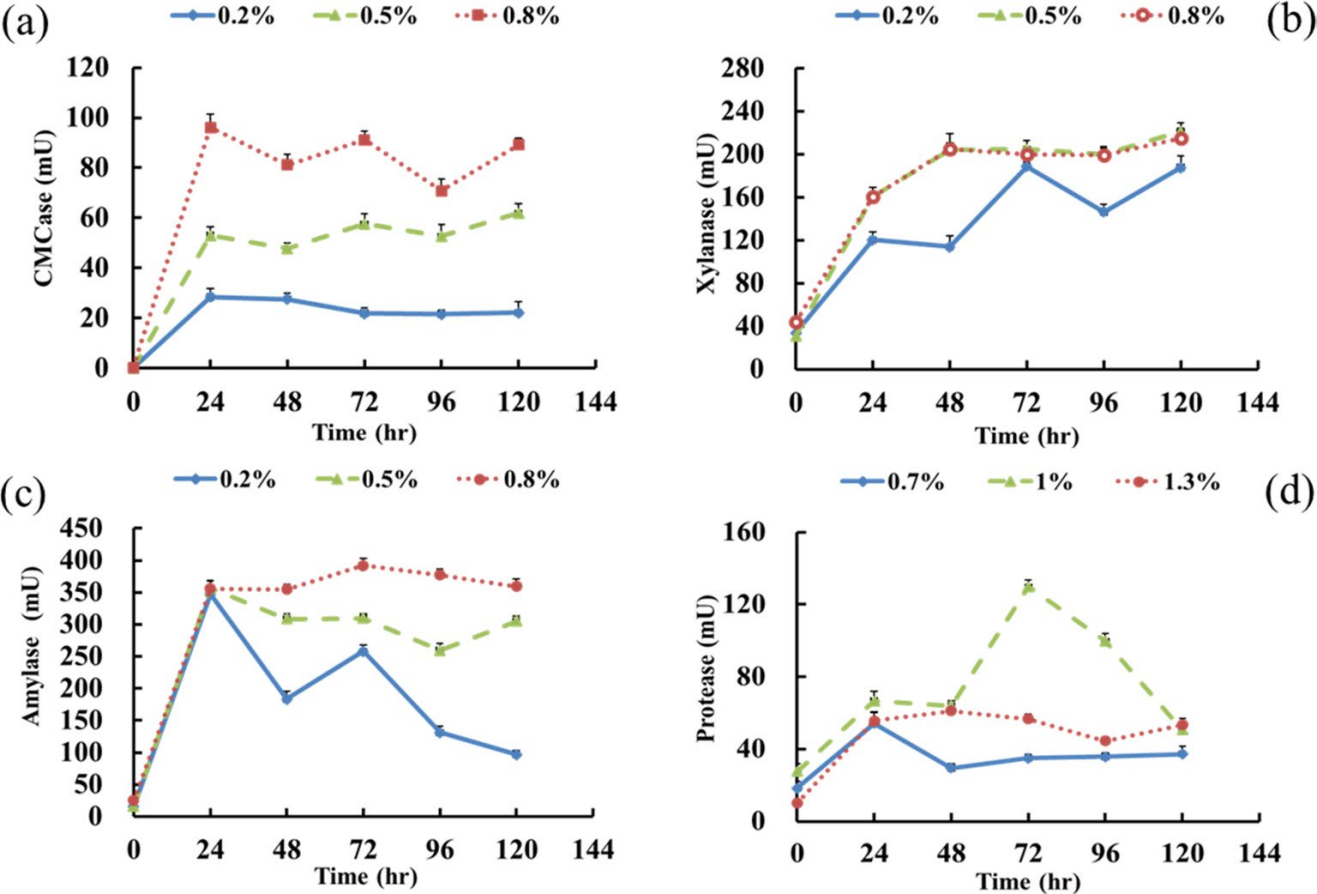
Changes in enzyme activity at different carbon source concentrations. (**a**) *Kitasatospora phosalacinea* C1, (**b**) *Paenibacillus glycanilyticus* X1, (**c**) *Bacillus licheniformis* S3, (**d**) *Brevibacillus agri* E4.

To understand the effect of carbon source concentration on the activities of enzymes, including CMCase, xylanase, amylase and proteinase, various carbon source concentrations (0.2%, 0.5% and 0.8%) were added at the beginning to evaluate the activity variation of target enzymes. The activity variation of target enzymes (CMCase, xylanase, amylase and proteinase) under different carbon source concentrations is displayed in Fig. 5. When *K. phosalacinea* strain C1 was cultured at 31 ℃ and an initial pH of 5.5, the CMCase activity was highest. The CMCase activities under three carbon source concentrations increased significantly within 24 h and remained stable after 24 h. The enzyme activity of the 0.8% carbon source was better than that of the 0.2% and 0.5% sources (Fig. 5a). Therefore, the optimal culture conditions for *K. phosalacinea* strain C1 to produce CMCase were 31 ℃, an initial pH of 5.5 and a carbon source concentration of 0.8%.

The environmental conditions with the best xylanase activity for *P. glycanilyticus* strain X1 were 45 ℃ and an initial pH of 7.5. The overall trend of the xylanase activity at a 0.2% carbon source concentration was lower than that of the other carbon source concentrations. In addition, although the xylanase activities of the 0.5 and 0.8% carbon source concentrations were similar, the xylanase activity of the 0.5% carbon source was slightly higher than that of the 0.8% carbon source (Fig. 5b). Thus, a carbon source concentration of 0.5% was suggested to be the optimal concentration for *P. glycanilyticus* strain X1.

Within 24 h, the amylase activities of the three carbon source concentrations increased over time. However, the amylase activity of the 0.8% carbon source was higher than that of the other concentrations after 24 h and remained stable until the end of the experiment (Fig. 5c). Therefore,to generate the highest amylase activity, the optimal incubation conditions for *B. licheniformis* strain S3 were 31 ℃, and an initial pH of 7.5 with a 0.8% carbon source concentration.

The optimal condition for the extracellular proteinase secreted by the protein-decomposing *B. agri* E4 was 45 ℃ and an initial pH of 8.5. Carbon source concentrations of 0.7%, 1% and 1.3% were applied under the optimal temperature and pH to understand the changes in proteinase activity (Fig. 5d). The proteinase activity of *B. agri* E4 at a 1% carbon source concentration was significantly higher than those of the others, and the highest proteinase activity reached 129.7 mU at 72 h (Fig. 5d).

### Composting tests

**Figure 6:**
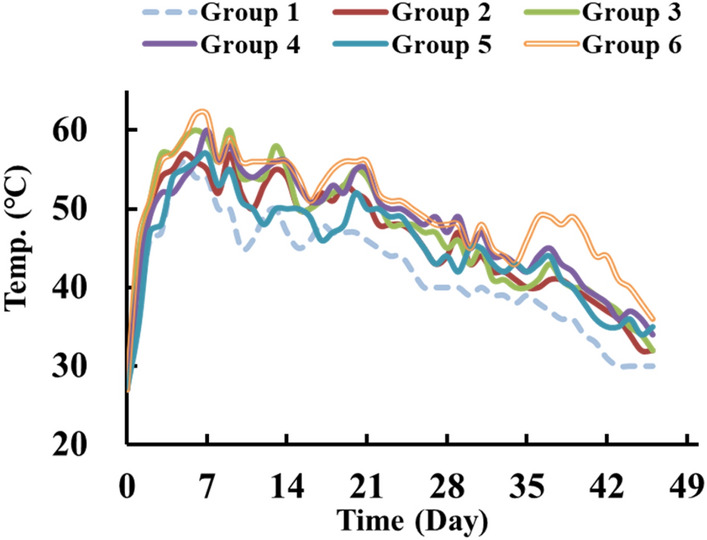
Temperature changes of different composting test groups.

Temperature is one of the important factors affecting microbial activity during composting and mainly affects the growth rate of microorganisms and speed of metabolic reactions. Figure 6 shows the temperature variation of different composting test groups during the composting process.

The results showed that the highest temperature in Group 1, which did not involve any microorganisms, was 56 ℃. This temperature was lower than that of other experimental groups inoculated with four bacterial isolates and one fungal strain, and the period in which the temperature was maintained above 50 ℃ was also the shortest. The temperature of Group 3 increased faster than that of the other test groups, and reached the maximum temperature of 60 ℃ on the 6th day. The temperature of Group 2 increased more slowly than that of Group 3 and reached 57 ℃ on day 5. Composting Group 4 reached the maximum temperature of 60 ℃ on day 7, and the temperature in the later period was slightly higher than that in the other test groups. For composting Group 5, the temperature increased relatively slowly and reached 57 °C on the 7th day. The temperature of Group 6 reached the maximum temperature of 62 ℃ on the 6th day. Additional inoculation was performed in Group 6 on day 30, when the composting process was in the cooling stage. The temperature of Group 6 increased again on the day 34. Yu et al. also demonstrated that the temperature rose again afterreinoculation of *P. chrysosporium* BKM-F-1767 at the cooling stage of 45 ℃ in composting soil containing straw, vegetables, bran and contaminated pentachloro-phenol^36^.

**Figure 7:**
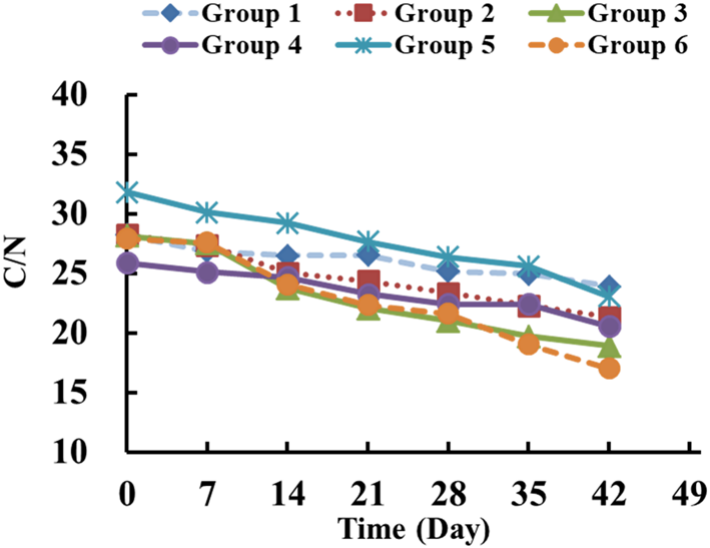
Change of C/N ratio during different test group during composting.

Figure 7 presents the changes in the C/N ratio of each composting group. The C/N ratio of each test group decreased as the composting time increased. Group 3 had the most appropriate initial C/N ratio among the 6 test groups. After composting for 42 days, the C/N ratio dropped from 28.14 to 18.93. The C/N ratio in Group 4 showed a slower downward trend, and it was similar to that of Group 2, which was only inoculated with *P. chrysosporium* strain. The final C/N ratios of Group 4 and Group 2 were 20.57 and 21.32, respectively. For Group 5, the initial C/N ratio of 30 was relatively high, and it decreased slowly to a final ratio of 23.09. Group 6 was reinoculated with microorganisms at the cooling stage, and the final C/N ratio was 17.03. All the results indicated that reinoculated could lead to further decrease in the C/N ratio in the composting process. The C/N ratio decreased most slowly in Group 1 because additional strains were not inoculated in the compost, and the final C/N ratio was 23.91. A previous study also demonstrated that reinoculation with *P. chrysosporium* BKM-F-1767 at the cooling stage (45 ℃) could shorten the time at which the C/N ratio decreased to 20 by 4 days, and this result was different from that observed with reinoculation at the mesophilic stage^36^. These results are also consistent with the results of this study. Zeng et al. inoculated *P. chrysosporium* strain AF 96007 into compost containing rice straw, vegetables, wheat bran and soil with an initial C/N ratio of 30. The lowest C/N ratio of 16 was obtained on day 42^37^. The C/N ratio decreased faster than that in this study, suggesting that the compost material in the literature study was highly biodegradable and that the added soil represented a type of inoculation, with both of these factors facilitating the compost maturation rate.

### Seedling growing test

To evaluate compost maturation, a seedling growing test was conducted in this study. Table 4 shows the seed germination efficiency of substrates obtained from each test group before and after composting. The results indicated that the seed germination efficiencies of Groups 1–6 were 56%, 68%, 52%, 60%, 56% and 40% for substrates that were obtained before composting. However, the seed germination efficiencies of Groups 1–6 increased to 76%, 84%, 96%, 84%, 80% and 92% for substrates obtained after composting. It was believed that the composting process was completed and reached maturity when the germination efficiency in the test group was greater than 90% and germinated root growth was not inhibited. Table 4 shows the percentages of test group to control group were 116%, 128%, 146%, 128%, 122% and 140% in the order of Group 1–6, respectively. Furthermore, the germinated root lengths before composting were 1.37 cm, 0.85 cm, 0.52 cm, 1.24 cm, 0.72 cm and 0.89 cm in the order of Group 1–6 whereas the lengths after composting increased to 2.33 cm, 2.72 cm, 3.43 cm, 2.90 cm, 2.09 cm and 2.94 cm in the order of Group 1–6. The results from the seedling growing test strongly demonstrated that all the test compost groups reached the mature stage. It was obvious that the period needed for compost maturation in Group 3 and Group 6 was significantly shorter than that in other test groups. Moreover, inoculation with four bacterial isolates and one fungal strain could improve the composting maturation.

**Table 4:** The seed germination rateefficiency of substrates obtained from each test group before and after composting.

Time (day)	Group 1	Group 2	Group 3	Group 4	Group 5	Group 6
	Seed germination rate (%)					
0	56	68	52	60	56	40
42	76	84	96	84	80	92
Test group /control group(%)	116	128	146	128	122	140

Time (day)	Germinated root length (cm)					
0	1.37	0.85	0.52	1.24	0.72	0.89
42	2.33	2.72	3.43	2.9	2.09	2.94

## Conclusions

Four bacterial isolates were obtained and identified as strains *K. phosalacinea*, *P. glycanilyticus*, *B. licheniformis* and *B. agri* after 16S rDNA sequencing, and they are responsible for degrading cellulose, xylan, starch and protein, respectively. The optimal culture conditioins of each isolate were characterized in this study. The proper initial C/N ratio was 27.5 for the coinoculation ofthe four bacterial isolates and one fungal strain (*P. chrysosporium*)*,* and this conidtion could facilitate increase in temperature and extend the thermophilic stage during the composting process. The C/N ratio decreased slowly when only the *P. chrysosporium* strain was added to the compost, although it could increase the rate of increase in temperature. Reinoculation with four bacterial isolates and the *P. chrysosporium* strain at cooling stage was beneficial to compost maturation. The results of this study indicate that the tested conditions represent a practical bioaugmentation method forenhancing compost maturation efficiency.
